# Nanomechanics of wild-type and mutant dimers of the inner-ear tip-link protein protocadherin 15

**DOI:** 10.1073/pnas.2404829121

**Published:** 2024-09-19

**Authors:** Camila M. Villasante, Xinyue Deng, Joel E. Cohen, A. J. Hudspeth

**Affiliations:** ^a^Laboratory of Sensory Neuroscience, The Rockefeller University, New York, NY 10065; ^b^Laboratory of Populations, The Rockefeller University, New York, NY 10065; ^c^Earth Institute, Columbia University, New York, NY 10027; ^d^Department of Statistics, Columbia University, New York, NY 10027; ^e^Department of Statistics, University of Chicago, Chicago, IL 60637; ^f^HHMI, The Rockefeller University, New York, NY 10065

**Keywords:** cochlea, entropic stiffness, gating spring, genetics of deafness, hair cell

## Abstract

Mechanical force controls the opening and closing of mechanosensitive ion channels atop the hair bundles of the inner ear. The filamentous tip link connecting transduction channels to the tallest neighboring stereocilium modulates the force transmitted to the channels and thus changes their probability of opening. Each tip link comprises four molecules: a dimer of protocadherin 15 (PCDH15) and a dimer of cadherin 23, all of which are stabilized by Ca^2+^ binding. Using a high-speed optical trap to examine dimeric PCDH15, we find that the protein’s mechanical properties are sensitive to Ca^2+^ and that the molecule exhibits limited unfolding at a physiological Ca^2+^ concentration. PCDH15 can therefore modulate its stiffness without undergoing large unfolding events under physiological conditions. The experimentally determined stiffness of PCDH15 accords with published values for the stiffness of the gating spring, the mechanical element that controls the opening of mechanotransduction channels. When PCDH15 exhibits a point mutation, V507D, associated with nonsyndromic hearing loss, unfolding events occur more frequently under tension and refolding events occur less often than for the wild-type protein. Our results suggest that the maintenance of appropriate tension in the gating spring is critical to the appropriate transmission of force to transduction channels, and hence to hearing.

## Significance

Our hearing is mediated by hair cells in the cochlea, each of which possesses a mechanosensitive hair bundle. Mechanical stimuli derived from sounds pull upon molecular filaments—the tip links—that open ionic channels in the hair bundle. Each tip link is a tetramer of filamentous cadherin proteins. Using an optical trap, we have exerted mechanical forces on individual dimers of one protein, protocadherin 15 (PCDH15). We find that a dimer is stable in the presence of a high Ca^2+^ concentration, but becomes less stable at a physiological level of the ion and quite unstable in its absence. Moreover, a mutation in PCDH15 that is associated with human deafness further destabilizes the protein.

**Fig. 1.**
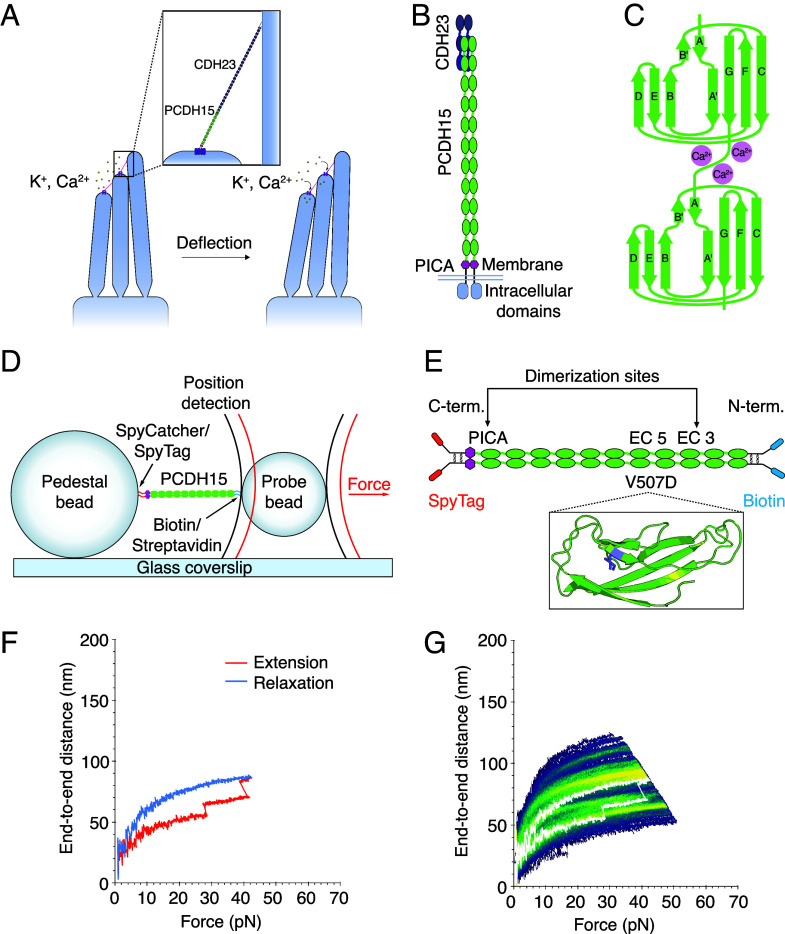
The structure of PCDH15 and measurements with an optical trap. (*A*) When a hair bundle is deflected toward its tall edge, the tip links (*Inset*) that interconnect the stereocilia stretch, opening the mechanotransduction channels (purple) atop each stereocilium and allowing cations in the endolymph to flow into the cell and depolarize it. (*B*) Each strand of a PCDH15 dimer comprises 11 EC domains and a PICA domain at its carboxy terminus. PCDH15 binds with CDH23 at their amino termini in a handshake interaction. (*C*) EC domains are composed primarily of β-sheets. Ca^2+^ binding in the linker regions and at the edges of the domains stabilizes the structure against unfolding. (*D*) In our apparatus, a dimeric protein molecule is tethered between two beads and two laser beams act on the probe bead to measure its position and exert force on it. (*E*) In our experimental construct, we maintain the dimerization sites in PCDH15 while adding two disulfide bonds and a distinct molecular tag at each end. The V507D construct is identical to the wild-type construct save for the insertion of the mutation in place of V507 (purple in *Inset*). (*F*) A force-ramp experiment comprises the extension phase of the cycle, during which force is increased at a constant rate, and the relaxation phase of the cycle, during which force is decreased back to a minimum at the same rate. In these experiments, the minimum force is 1 pN and 2 s elapses between successive cycles. Unfolding events can be seen as sudden steps during an individual extension. (*G*) Repetition of a force-ramp cycle hundreds of times on the same protein molecule yields a heat map in which the brighter colors represent more highly occupied states. The heatmap superimposes both extension and relaxation phases of every cycle. The illustrative record from panel *F* is overlaid in white.

The transformation of sound waves into neural signals, a process known as mechanoelectrical transduction, occurs in the cochlea of the internal ear. To allow humans to hear with the range and precision that we do, this process must be both sensitive and adaptive. The apical surface of each sensory hair cell bears actin-filled stereocilia arranged in order of height. Each stereocilium is connected by a filamentous tip link to its tallest neighbor (1). When the hair bundle is deflected toward its tall edge by a sound stimulus, the stereocilia pivot and tense the gating springs that control the opening of the mechanoelectrical transduction channels atop each stereocilium. When sufficient tension has been conveyed, the channels open and allow cations from the endolymph surrounding the hair bundles to flow into the cells and transduction to occur (2, 3) (Fig. 1*A*).

It is plausible that the gating springs are the filamentous tip links that connect the transduction channels of a stereocilium to its tallest neighbor. Each tip link comprises four protein molecules (4): a dimer of protocadherin 15 (PCDH15) and a dimer of cadherin 23 (CDH23). Because abolishing the tip links halts mechanotransduction (5) whereas allowing the tip links to regenerate restores sensitivity (6), the tip link—or the attachments at its two ends—likely controls the opening and closing of the channels and serves as the gating spring. Moreover, the hundreds of mutations in tip-link proteins that result in human hearing loss (7–11) underscore the role of the tip link in hearing.

The direct association of PCDH15 with the transduction–channel complex by way of its transmembrane and cytoplasmic domains (12) implicates the protein in channel gating. PCDH15 comprises 11 extracellular cadherin (EC) domains and a PCDH15-interacting, channel-associated (PICA) domain, also known as MAD12 (13) or EL (12) (Fig. 1*B*). The EC domains, which are composed of Greek key motifs, are similar in their folding patterns (Fig. 1*C*). The intervening linker regions can bind up to three Ca^2+^ ions (13) and stabilize the entire molecule against unfolding by force (14). The concentration of Ca^2+^ in the cochlear endolymph is approximately 20 μM, a value much lower than that in the rest of the body (15). The dissociation constant of Ca^2+^ at the linker regions lies in the range of tens to hundreds of micromolar (13), a range that might confer additional modulation of PCDH15 behavior at physiological Ca^2+^ concentrations.

Depending on the local characteristic frequency and whether measured in inner or outer hair cells, the enthalpic stiffness of gating springs in the rat’s cochlea (16) lies between 0.5 mN m^−1^ and 4 mN m^−1^. Although monomers of PCDH15 have an enthalpic stiffness of approximately 10 mN m^−1^, the measured stiffness of monomeric PCDH15 is lower over the physiological range of forces (17). This softening represents the contribution of entropic elasticity, which stems from the extension of the interdomain linker regions, the EC domains themselves, and any unfolded portions of the molecules. We inquired whether dimeric PCDH15 would exhibit a similar softening over the physiological range of forces and whether its enthalpic stiffness would accord with that of the gating spring. To understand whether PCDH15 has the appropriate properties to be a component of the gating spring, we measured its stiffness, inquired what factors control its mechanical responses, and examined how it softens as a result of conformational changes when under tension.

## Results

### Experimental Conditions.

To probe the mechanical properties of PCDH15 directly, we used an optical trap with subnanometer spatial resolution and microsecond temporal resolution (17, 18) (Fig. 1*D*). In a typical experiment, PCDH15 was tethered between two beads: At its carboxy terminal, it was attached by a covalent SpyTag-SpyCatcher bond (19) to a pedestal bead covalently attached to a glass coverslip, and at its amino terminal it was linked through a biotin–streptavidin interaction to a probe bead in solution (Fig. 1*E* and *SI Appendix, Materials and Methods*). The anchors that attached PCDH15 to the beads on either end were separated from the protein by short, unstructured peptides (*SI Appendix*, Fig. S1). Two lasers acted on the probe bead: a highly stable 1,064 nm position-sensing laser, which detected the three-dimensional position of the bead and thus the extension of the construct, and an 852 nm force-producing laser, which exerted force on the bead and therefore on the protein.

To explore the range of physiological forces that PCDH15 experiences in the ear (20), we conducted force-ramp experiments in which force was increased at a constant rate from a resting level of 1 pN and then decreased at the same rate back to the resting level, at which it was held for 2 s before the next cycle (*SI Appendix*, Fig. S3). During these extension-relaxation cycles, the protein sometimes underwent large, rapid conformational changes. Such unfolding events could be seen as steps in the end-to-end distance (Fig. 1*F*). After a force-ramp cycle had been repeated up to hundreds of times on a single protein molecule, all the cycles could be displayed as a heatmap in which lighter colors correspond to more frequently occupied states and darker colors represent trajectories that occurred only once or infrequently (Fig. 1*G*).

Because of the importance of Ca^2+^ to the structure of PCDH15, we performed our experiments at three representative Ca^2+^ concentrations: 3 mM, a saturating level meant to populate all the Ca^2+^-binding sites; 20 μM, a physiological concentration in the endolymph of the mammalian cochlea (21–23); and with no Ca^2+^, but in the presence of the Ca^2+^ chelator ethylenediaminetetraacetic acid (EDTA). Previous studies on monomeric PCDH15 showed that its mechanical behavior changes with the Ca^2+^ concentration (17), so we explored whether the dimer shows a similar Ca^2+^ dependence.

### Ca^2+^ Sensitivity of PCDH15 under Physiological Forces.

PCDH15 naturally dimerizes at EC3 and the PICA domain (24). To ensure that force was distributed equally to both strands of each PCDH15 dimer, we devised a construct in which the monomers were attached to one another at each end by paired disulfide bonds derived from the Fc hinge region of human immunoglobulin (25) (Fig. 1*E*). This study used murine PCDH15, which has high sequence homology (26) to human PCDH15.

**Fig. 2.**
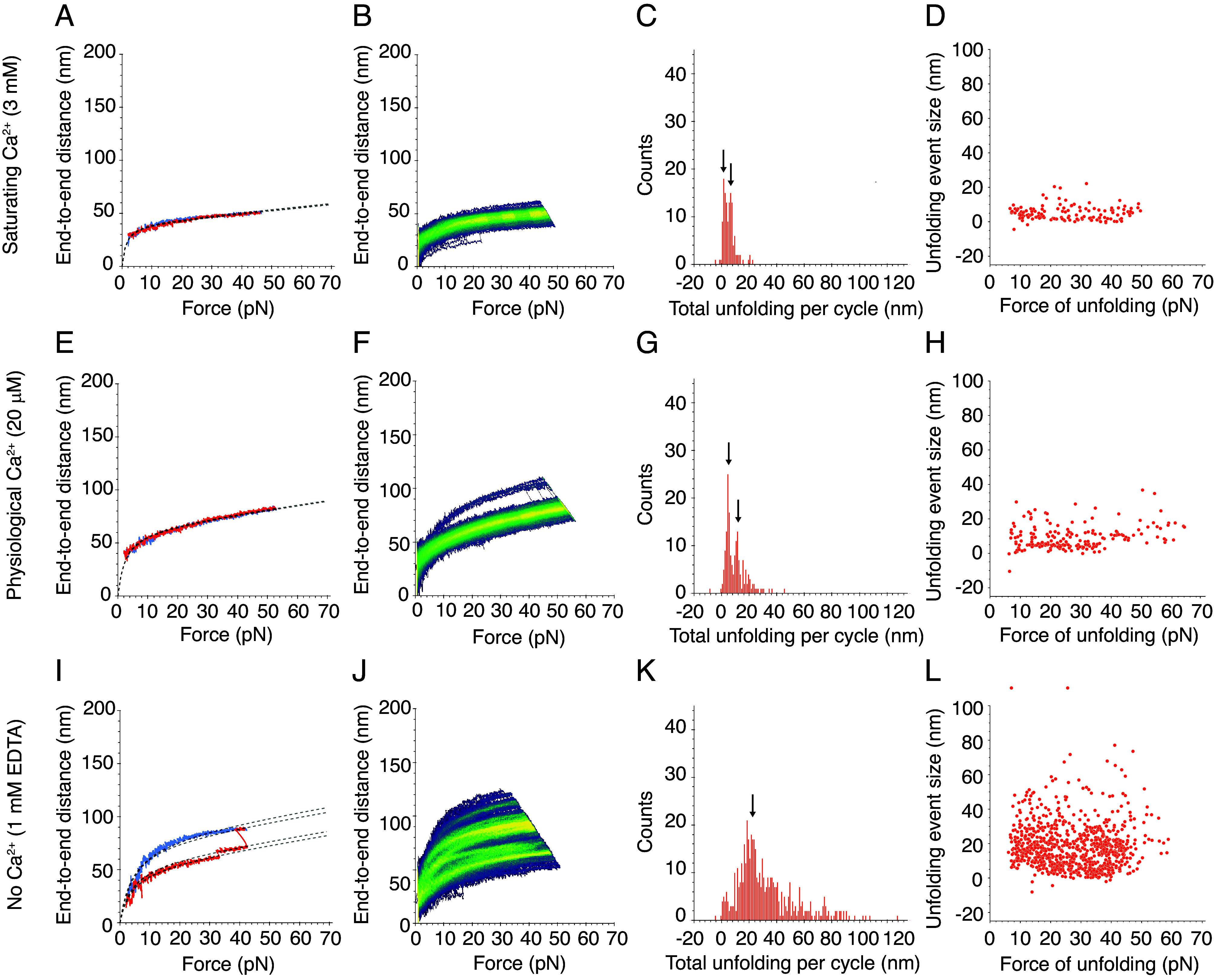
Force-ramp responses of wild-type PCDH15 at three Ca^2+^ concentrations. (*A*) At a saturating Ca^2+^ concentration of 3 mM, PCDH15 unfolded infrequently. The dashed line represents a fit of Eq. **1** to the extension and relaxation phases of the illustrative cycle. In a fit of Eq. **1** to the extension phase of the cycle, *x_E_* = 39.9 nm and *F_HALF_* = 1.4 pN. In a fit to the relaxation phase, *x_E_* = 40.9 nm and *F_HALF_* = 1.5 pN. For both phases, *K* = 3.7 mN m^−1^. (*B*) In a heatmap for a saturating level of Ca^2+^, 3 mM, the single bright branch indicates one highly occupied state that reflects the infrequent and small unfolding events. (*C*) The total unfolding length during the extension phases of all cycles in all datasets at 3 mM Ca^2+^ peaked at 1.8 ± 0.1 nm and 6.3 ± 0.2 nm (arrows; means ± SEMs; *N* = 5 datasets; *n* = 131 events). (*D*) Many unfolding events occurred at forces below 10 pN at 3 mM Ca^2+^. (*E*) At a physiological Ca^2+^ concentration of 20 μM, PCDH15 often did not unfold and was more extensible than with 3 mM Ca^2+^ in the same force range. In a fit of Eq. **1** to the extension phase of the cycle, *x_E_* = 63.8 nm and *F_HALF_* = 2.4 pN. In a fit to the relaxation phase, *x_E_* = 63.1 nm and *F_HALF_* = 2.4 pN. For both phases, *K* = 2.5 mN m^−1^. (*F*) The illustrative heatmap has one bright branch indicative of the infrequent and small unfolding events at 20 μM Ca^2+^. (*G*) At 20 μM Ca^2+^, the total unfolding per cycle was slightly greater than at a saturating level of Ca^2+^, with peaks at 4.5 ± 0.1 nm and 11.5 ± 0.1 nm (arrows; means ± SEMs; *N* = 4 datasets; *n* = 172 events). (*H*) At 20 μM Ca^2+^, individual unfolding events with a mean of 4.6 ± 0.1 nm occurred predominantly at forces below 40 pN. (*I*) An illustrative force-ramp cycle in the absence of Ca^2+^ and in the presence of 1 mM EDTA shows a small unfolding event followed by a larger one. In this case, Eq. **1** is fitted separately to each segment demarcated by the unfolding events. In a fit to the segments in the extension phase of the cycle, *F_HALF_* = 4.1 pN, whereas *x_E_* = 55.2 nm, 59.6 nm, and 78.8 nm for the successive segments. In a fit to the relaxation phase, *x_E_* = 84.5 nm and *F_HALF_* = 5.1 pN. For both phases, *K* = 2.4 mN m^−1^. (*J*) The numerous bright branches in the heatmap reflect the multiple preferred conformational states of PCDH15 in the absence of Ca^2+^ and in the presence of 1 mM EDTA. (*K*) The total unfolding per cycle peaked at 23.3 ± 0.6 nm (arrows; mean ± SEM; *N* = 6 datasets, *n* = 490 events), but many larger events occurred in the absence of Ca^2+^ and in the presence of 1 mM EDTA. (*L*) There is no clear relationship between the size of individual unfolding events and the corresponding forces in the absence of Ca^2+^ and presence of 1 mM EDTA.

As exhibited both by a representative cycle (Fig. 2*A*) and by the single bright branch on a representative heatmap (Fig. 2*B*), dimeric PCDH15 underwent little unfolding at a saturating level of Ca^2+^. At low forces, PCDH15 extended easily in response to applied force, a behavior that reflects entropic elasticity owing to the straightening of the molecule’s thermal undulations. At higher forces, after most entropic degrees of freedom had been pulled out, the relationship between the end-to-end distance and the applied force became nearly linear. The remaining extensibility resulted from the enthalpic or Hookean stiffness of PCDH15.

To quantify and classify the unfolding changes that occurred, we used a saturation model augmented with a linear-spring term: If *x* is the end-to-end distance of the PCDH15 construct, and *F* is the applied force,

[1] $$x=\frac{x_{E}}{1+\frac{F_{\mathrm{HALF}}}{F}}+\frac{F}{K}.$$

The maximal entropic extension of PCDH15 is given by *x*_E_; *F*_HALF_ is the force at which entropic extension is halfway complete. The contribution of enthalpic stiffness is given by the second term, the extension of a linear spring of stiffness *K* under force *F*. The value of *K* was determined by averaging the inverse spatial derivatives at forces exceeding 30 pN for every cycle of all datasets at each Ca^2+^ concentration.

At a saturating concentration of Ca^2+^, 3 mM, few discrete unfolding events occurred, and their magnitudes were relatively small. The frequency distribution of the size of these unfolding events was bimodal with peaks at 2.0 nm and 6.6 nm (*SI Appendix*, Fig. S4*A*). We asked whether these individual unfolding events happened in succession within the same cycle, which would suggest the occurrence of a larger unfolding event through a multistep process. We summed the total unfolding lengths in the extension phase of each cycle, during which the majority of unfolding events occurred. The total unfolding length per cycle was predominantly below 20 nm, with a bimodal frequency distribution peaking at 1.8 nm and 6.3 nm (Fig. 2*C*). These events could correspond to extension of the interdomain linker regions, which—because they lack secondary structure—are likely to be the first components of PCDH15 to unfold under force. Individual linker regions would give rise to an additional length between 1 nm and 2 nm when extended, so it is plausible that the unfolding of linker regions yielded the unfolding events that we observed. Furthermore, not all the linker regions bind three Ca^2+^ ions—some bind none, whereas others bind one or two ions (13)—so we might have observed extension of these linker regions even at a saturating level of Ca^2+^. It is also possible that the small extensions reflected the straightening of the kinked EC9-10 linker (13, 27), which does not bind Ca^2+^ and could extend up to 4 nm. We also asked whether there was any relationship between the size of individual unfolding events and the force at which they occurred. However, we found no clear relationship between the size of unfolding events and the force of unfolding (Fig. 2*D*).

PCDH15 also unfolded infrequently at a physiological Ca^2+^ concentration of 20 μM. Nevertheless, at that concentration PCDH15 was strikingly more extensible than at a saturating level of Ca^2+^ (Fig. 2 *E* and *F*). In other words, despite the scarcity of unfolding events, the dimer was softer at the lower concentration of Ca^2+^. This finding is surprising, for we had expected an increase in compliance to emerge through more frequent unfolding events. The observation might reflect the differential Ca^2+^-binding behaviors of the linker regions in PCDH15: The heterogeneity in the linkers, combined with the relatively low physiological concentration of Ca^2+^ and the binding affinity for Ca^2+^, might result in the overall softening. As was observed at a saturating Ca^2+^ concentration, the frequency distribution of total unfolding length per cycle was bimodal with peaks at 4.5 nm and 11.5 nm (Fig. 2*G*). These values are similar in magnitude to the peaks of total unfolding length per cycle that we observed at a saturating level of Ca^2+^. It was not possible to discern the origin of these classes of unfolding events owing to the repetitive structure of PCDH15. However, from the magnitudes of the conformational changes and assuming a length of 0.40 nm per amino acid (28), we conjecture that these two classes arose from the interdomain linker regions of PCDH15 that, when unfolded, would have given rise to a total of 16 nm of additional length. The individual unfolding events were again distributed across a range of forces (Fig. 2*H*). These results suggest that, at saturating and physiological Ca^2+^ conditions, extension of the linkers confers flexibility to PCDH15 and modulates its stiffness.

In the absence of Ca^2+^, PCDH15 underwent many types of unfolding that can be seen both in individual cycles and in numerous highly occupied branches on the heatmap (Fig. 2 *I* and *J*). There were far more unfolding events than were observed at higher Ca^2+^ concentrations. The sum of the lengths of unfolding events per cycle, although peaking in frequency at 23.3 nm (Fig. 2*K*), extended to values above 100 nm. We would expect the unfolding of a full-length cadherin or PICA domain to extend the end-to-end length of PCDH15 by 33 nm to 45 nm, depending on the particular domain that unfolded (29), and assuming a length of 0.40 nm per amino acid (28) less 4.5 nm to account for the loss of the folded domain (30). Because we do not observe a clear peak in that range, we infer that the unfolding of full domains was not the primary response to applied force. The most commonly observed length of unfolding could correspond to partial domain unfolding: If the A and B strands of an individual EC domain were to come undone, the end-to-end distance would increase by approximately 17 nm. Especially because of its association (12, 31) with the protein LHFPL5, it is less clear how a PICA domain might unfold incompletely. In experiments, the individual unfolding events occurred over a range of forces, with no clear relationship between the size of the unfolding event and the force at which it occurred (Fig. 2*L*). This result is surprising, for we had expected to see more frequent domain unfolding in the absence of Ca^2+^, but our results suggest that an alternative mechanism prevails.

### Effects of a Hearing-Loss Mutation on PCDH15.

Numerous mutations in the tip link result in hearing loss (7, 8, 11, 32). Such mutations can cause either syndromic deafness, in which deafness is accompanied by other deficits such as blindness or vestibular dysfunction, or nonsyndromic deafness, which involves exclusively hearing loss. Though PCDH15 is present in the retina and vestibular labyrinth as well as the cochlea, individuals with nonsyndromic deafness retain normal retinal and vestibular function: Only their hearing is affected, which raises interesting questions about the pathophysiology of these mutations.

We sought to understand how a deafness-causing mutation affects the mechanics of PCDH15. We chose to study V507D, the murine homolog of the human V528D variant that is associated in humans with nonsyndromic, autosomal, recessive deafness type DFNB23 (32). This point mutation of a highly conserved valine in EC5 was identified in a Newfoundland family whose members exhibit prelingual hearing loss. The V507 residue occurs in the β-sheet of the B strand of EC5 (Fig. 1*E*), which is neither a site of dimerization, nor part of the handshake interaction with CDH23, nor a Ca^2+^-binding site, any of which would result in obvious disruptions of tip link integrity. We then used AlphaFold2 Colab (33–37) to predict the structures of the EC5 domains in wild-type and V507D molecules. Alignment of the crystal structure and AlphaFold2-predicted wild-type EC5 showed that the prediction was highly accurate (*SI Appendix*, Fig. S5*A*). The predicted structure of V507D EC5 indicates that the β-sheet structure of strand B would be disrupted by substitution of the negatively charged aspartic acid residue (*SI Appendix*, Fig. S5 *B* and *C*). This alteration could result in an easier unfolding of strands A and B, which in the native protein are stabilized by a parallel β-sheet interaction between strands A and G and a stronger antiparallel β-sheet interaction between strands B and E (Fig. 1*C*). Disruption of the β-sheet interaction of strands B and E could therefore cause an instability of strand B. We conjecture that the change of this hydrophobic valine to a negatively charged aspartic acid weakens the force-bearing ability of the β-sheet and thus compromises the structural integrity of EC5. Our AlphaFold2 predictions of the aspartic acid substitution at position 507 show the side chain pointing outward in order to accommodate the negative charge, a distinct alteration from the wild-type valine side chain that points toward the hydrophobic core of the domain (*SI Appendix*, Fig. S5 *B* and *C*). The mutation could both disrupt the β-sheet structure and expose its hydrophobic core. Furthermore, the EC5-6 linker region binds only one Ca^2+^ ion, which might predispose EC5 to unfolding.

**Fig. 3.**
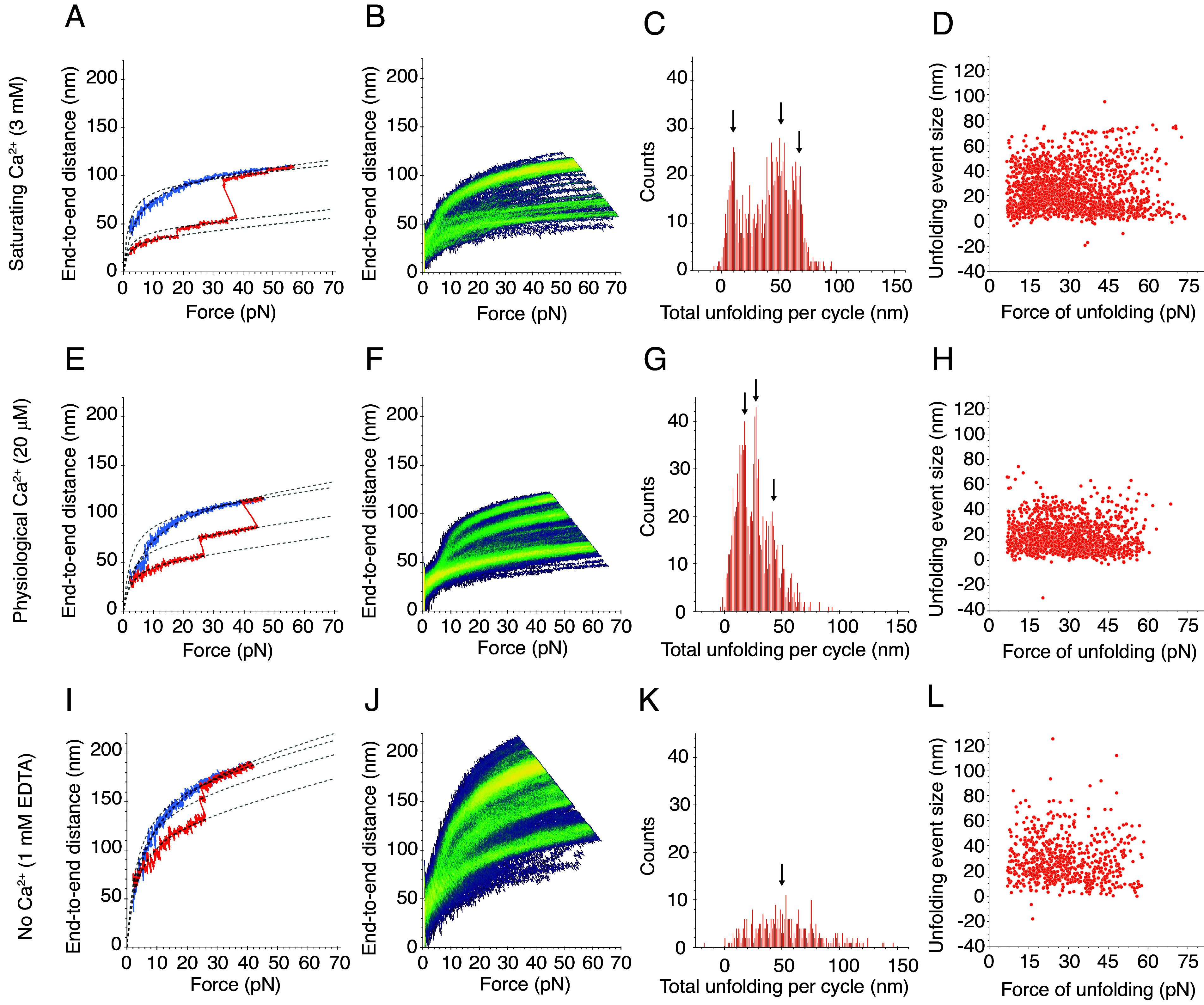
Force-ramp responses of V507D at three Ca^2+^ concentrations. (*A*) At a saturating level of Ca^2+^, 3 mM, a subset of V507D molecules underwent only small unfolding events, whereas another subset had more frequent unfolding (*SI Appendix*, Fig. S5*A*). The dashed line represents the fit of Eq. **1** to the segmented extension and relaxation phases. In a fit to the segments in the extension phase of the cycle, *F_HALF_* = 2.4 pN, whereas *x_E_* = 34.9 nm, 44.0 nm, and 91.6 nm for the successive segments. In a fit to the relaxation phase, *x_E_* = 99.5 nm and *F_HALF_* = 4.0 pN. For both phases, *K* = 3.2 mN m^−1^. (*B*) The bright branches on the heatmap reflect the frequent unfolding events observed in individual cycles. (*C*) The frequency distribution of total unfolding length per cycle peaked around 8.8 ± 0.1 nm, 50.0 ± 0.2 nm, and 66.3 ± 0.1 nm (means ± SEMs; *N* = 24 datasets; *n* = 1,889 events). (*D*) As the size of the individual unfolding events grew larger, the force of unfolding declined, with many events occurring around 30 pN. (*E*) At a physiological concentration of Ca^2+^, 20 μM, frequent unfolding was seen at the single-cycle level. In a fit of Eq. **1** to the segments in the extension phase of the cycle, *F_HALF_* = 2.1 pN, whereas *x_E_* = 48.1 nm, 68.6 nm, and 98.6 nm for the successive segments. In a fit to the relaxation phase, *x_E_* = 110.9 nm and *F_HALF_* = 6.4 pN. For both phases, *K* = 2.1 mN m^−1^. (*F*) The unfolding seen in the individual cycles is reflected by the bright branches on the exemplary heatmap. (*G*) The frequency distribution of total unfolding length per cycle during the extension phase peaked around 14.2 ± 0.2 nm, 27.3 ± 0.1 nm, and 39.4 ± 0.3 nm (means ± SEMs; *N* = 16 datasets; *n* = 1,081 events). (*H*) As at a saturating level of Ca^2+^, the larger unfolding events were associated with smaller forces of unfolding. (*I*) When Ca^2+^ was absent, V507D extended easily, even without large unfolding events. In the fit of Eq. **1** to the segments in the extension phase of the cycle, *F_HALF_* = 3.7 pN, whereas *x_E_* = 130.9 nm, 157.6 nm, and 174.0 nm for the successive segments. In a fit to the relaxation phase, *x_E_* = 188.2 nm and *F_HALF_* = 6.2 pN. For both phases, *K* = 1.6 mN m^−1^. (*J*) The bright branches on the illustrative heatmap reflect the unfolding behavior seen on the individual cycle level. The upper limits of the end-to-end distance at this level of Ca^2+^ exceeded those seen at higher concentrations of Ca^2+^. (*K*) The frequency distribution of total unfolding length per cycle was unimodal, with one peak at 49.3 ± 1.4 nm (mean ± SEM; *N* = 4 datasets; *n* = 362 events). (*L*) In the absence of Ca^2+^, there was no clear relationship between the size of individual unfolding events and the forces at which they occurred.

As the concentration of Ca^2+^ decreased from saturating to absent, PCDH15 V507D exhibited a striking pattern of increasing dysregulation. At a high Ca^2+^ concentration of 3 mM, and in contradistinction to the wild-type dimer, there were two populations of V507D molecules. Some V507D molecules behaved more like wild-type PCDH15 at the same saturating level of Ca^2+^, with the minimal unfolding observed in individual cycles reflected by a single bright branch on the heatmap (*SI Appendix*, Fig. S6). Other V507D molecules exhibited more unfolding during individual cycles, revealed by multiple prominent branches on the heatmap (Fig. 3 *A* and *B*). We included both kinds of force-extension trajectories in further analysis, for the tethering statistics (*SI Appendix, Note S1*) gave us confidence that specific trajectories corresponded to individual molecules rather than multiple tethers, and we observed both kinds of trajectories often enough to deem them significant.

Considering both populations, we observed many more unfolding events per cycle from V507D than from wild-type PCDH15 at a saturating concentration of Ca^2+^, for which we largely observed only small unfolding events below 10 nm in magnitude. Because the numerous unfolding events in the mutant protein occurred at a Ca^2+^ level likely to saturate Ca^2+^ binding sites in the interdomain linker regions, they likely stemmed from instability in EC5, where the mutated residue was located. The frequency of total unfolding length per cycle peaked at 8.8 nm, 50.0 nm, and 66.3 nm (Fig. 3*C*). The 50.0 nm unfolding events exceeded the size expected for the unfolding of an entire EC domain or the PICA domain, 33 to 45 nm, but are smaller than expected for the unfolding of two domains. This suggests that some intermediate unfolding event gave rise to this class. The 66.3 nm class of events might reflect the unfolding of two domains, which would yield an end-to-end distance increase of 66 to 90 nm. Many unfolding events occurred at forces around 30 pN (Fig. 3*D*), suggesting that V507D has a diminished ability to resist applied force compared to the wild-type protein, which exhibits a more uniform distribution of unfolding forces.

At a physiological Ca^2+^ concentration of 20 μM, PCDH15 V507D again underwent unfolding events of varying magnitudes (Fig. 3 *E* and *F*). This behavior contrasted with that of the native protein at the same Ca^2+^ concentration, for which very few, small unfolding events were seen. The frequency distribution of total unfolding length within each cycle peaked at 14.2 nm, 27.3 nm, and 39.4 nm (Fig. 3*G*). Because the V507D mutation occurs in EC5, that domain is the most likely to unfold. If EC5 were to unfold and its neighboring linker regions to extend, we would expect an increase in end-to-end distance of 39.1 nm. The class at 39.4 nm therefore might well correspond to the complete unfolding of EC5. It is unclear what gave rise to the smaller classes of extension, though denaturation of the linker regions, which could result in a total 16 nm increase in end-to-end distance, might contribute. Although we predicted that V507D would be more unstable and exhibit more unfolding at this level of Ca^2+^, the unfolding lengths did not reach such high magnitudes as at a saturating Ca^2+^ concentration. This result suggests that V507D was extended more than the wild-type protein prior to the application of force. The partial unfolding at baseline implies that the unfolding we observed after applying force occurs in addition to the baseline unfolding, which explains the unexpected difference in the range of unfolding forces between physiological and saturating levels of Ca^2+^ (Fig. 3*H*).

Like the wild-type dimer, V507D underwent numerous unfolding events in the absence of Ca^2+^ as evidenced by the illustrative cycle and multiple bright branches on the illustrative heat map (Fig. 3 *I* and *J*). However, the bright branch closest to the origin—which reflects the extension of the protein in the absence of any unfolding events—extended to approximately 100 nm at the highest applied forces, whereas for the wild-type protein this branch reached only 50 nm. In addition, the largest end-to-end distances achieved for V507D exceeded 200 nm, a distance greater than the 125 nm characteristic of the wild-type molecule. In many individual cycles, we did not observe drastic unfolding events (Fig. 3*I*). Instead, the molecule appeared quite extended at baseline and lengthened easily with the application of force. This result suggests that the protein was already unfolded, or misfolded, at this concentration of Ca^2+^ even in the absence of force.

In the absence of Ca^2+^, although sometimes as much as 150 nm unfolded in a single cycle for V507D, the total unfolding length varied widely and the frequency peaked at 49.3 nm (Fig. 3*K*). The average total unfolding per cycle suggests that at least one full domain unfolded during the extension phase of a cycle. At this concentration, we were likely observing unfolding events similar to those at the higher levels of Ca^2+^, along with additional events stemming from the complete lack of Ca^2+^ binding at the linker regions. There was again no clear relationship between the sizes of unfolding events and the forces at which they occurred, but many unfolding events of different sizes occurred around 30 pN (Fig. 3*L*). Although by these metrics the behavior of V507D in the absence of Ca^2+^ resembled that at higher concentrations of Ca^2+^, the end-to-end distance range of the protein exceeded that at other Ca^2+^ levels. This result suggests that, in the absence of Ca^2+^, V507D did not refold properly between extension cycles, but was already extended at the resting force of 1 pN. Because V507D displayed behavior that was not entirely captured by our modeling approach, we next sought a method of classifying the extension patterns across different experimental conditions.

Because PCDH15 remained extended under a declining tension during the relaxation phase of each cycle, some unfolding events occurred during that phase as well. In general, the frequency of unfolding events was lower than that for the extension phase (*SI Appendix*, Fig. S7). Moreover, the magnitude of unfolding events was lower in the relaxation than the extension phase (*SI Appendix*, Fig. S8). The forces at which unfolding events transpired during the relaxation phase resembled those for the extension phase (*SI Appendix*, Fig. S9).

### Clustering of Data by Conformational State.

**Fig. 4.**
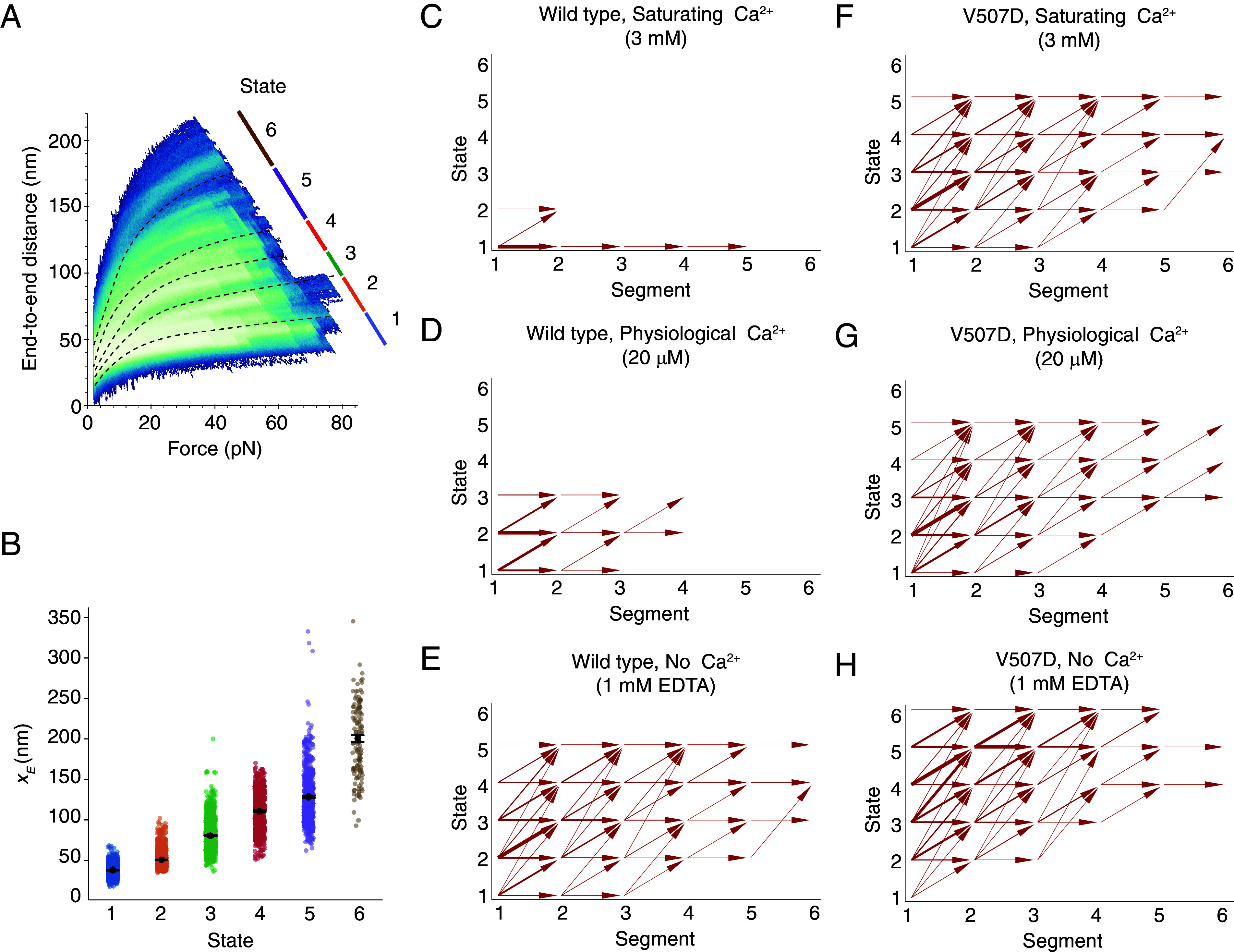
Clustering of force-ramp trajectories and interstate transitions. (*A*) In a heatmap representing the relaxation-phase trajectories from all Ca^2+^ conditions and both PCDH15 constructs, the lines with different colors demarcate six conformational states. (*B*) A scatter plot shows the fitted *x_E_* values for individual trajectories through different conformational states. The data represent means ± SEMs. (*C*–*H*) Conformational transition maps summarize the trajectories of the two PCDH15 constructs at different Ca^2+^ concentrations. Segmented by conformational changes, the values along the abscissa denote individual segments within a trajectory in ascending order of force. The thickness of each arrow’s shaft is proportional to the frequency of a specific interstate transition. The horizontal arrows indicate conformational changes within a state, whereas the arrows pointing up and to the right denote conformational changes made between distinct states.

To understand PCDH15 in various concentrations of Ca^2+^ with or without the V507D mutation, we analyzed all results from each construct and condition (*SI Appendix, Notes S2–S7*). The data fell into six classes, which we term “states” because we believe they represent distinct conformations of PCDH15 (Fig. 4*A* and *SI Appendix*, Fig. S10). The defining characteristics of each state are the average value of its *x_E_* parameter and the difference between that value and those of the contiguous states (Fig. 4*B*). Notwithstanding the substantial overlap in *x_E_* values of trajectories that we classify as belonging to different states, the average *x_E_* values clearly differ. For example, the difference between the average *x_E_* values for the first two states is 12.5 ± 0.3 nm (mean ± SEM; *n =* 2,314 trajectories for state 1, *n =* 1,826 trajectories for state 2), a distance consistent with the range of extension expected from the interdomain linker regions. Because the difference between states 1 and 2 is smaller than we would expect for the unfolding of an entire EC cadherin, we call states 1 and 2 the folded states—that is, the states in which all EC domains and the PICA domain apparently remain folded. The differences between states 2 and 3 and between states 3 and 4 are respectively 29.9 ± 0.7 nm (mean ± SEM, *n =* 1,372 trajectories for state 3) and 30.0 ± 1.3 nm (mean ± SEM, *n =* 692 trajectories for state 4), values consistent with the expectation for unfolding of a complete EC or PICA domain. The difference between states 4 and 5 is 17.6 ± 2.0 nm (mean ± SEM, *n =* 585 trajectories for state 5), a value that does not accord with unfolding of a whole domain but might represent partial unfolding of a domain. Finally, the difference between states 5 and 6 is 72.2 ± 6.0 nm (mean ± SEM, *n =* 123 trajectories for state 6), a value similar to that expected for the unfolding of two entire EC domains or one EC domain and the PICA domain.

To understand the effect of the Ca^2+^ concentration and V507D mutation on how PCDH15 traversed the accessible state space, we segmented each force-ramp trajectory by the conformational changes that occurred (*SI Appendix, Note S2*) and assigned each segment to a state through *k*-nearest-neighbor classification (*k* = 3; *SI Appendix, Note S4*). To generate transition maps for each construct and condition, we analyzed the fraction of time that a molecule spent in each state and examined the states visited in subsequent segments within one cycle (*SI Appendix*, Fig. S11). In saturating Ca^2+^ conditions, the wild-type protein remained predominantly within state 1, with a few excursions to state 2 (Fig. 4*C* and *SI Appendix*, Figs. S12*A* and S13*A*). At a physiological Ca^2+^ concentration, the native dimer spent much more time in state 2 and occasionally visited state 3, a behavior suggestive of unfolding of linker regions and one EC or PICA domain (Fig. 4*D* and *SI Appendix*, Figs. S12*C* and S13*A*). In the absence of Ca^2+^, the wild-type dimer progressed up to state 5, with cycles beginning primarily in state 2 or above rather than in state 1 (Fig. 4*E* and *SI Appendix*, Figs. S12*E* and S13*A*). Under this condition, entire EC domains were likely unfolding.

In contrast to the wild-type dimer in saturating Ca^2+^ conditions, V507D at the same Ca^2+^ level reached state 5 (Fig. 4*F* and *SI Appendix*, Figs. S12*B* and S13*B*). At a physiological Ca^2+^ level, V507D visited up to state 5 and exhibited transition behaviors similar to those in the saturating Ca^2+^ condition (Fig. 4*G* and *SI Appendix*, Figs. S12*D* and S13*B*). In the absence of Ca^2+^, V507D spent little time in states 1 and 2 and frequently started in higher states (Fig. 4*H* and *SI Appendix*, Figs. S12*F* and S13*B*), such as state 3, in which one EC domain had apparently unfolded already. The V507D dimer in the absence of Ca^2+^ favored higher states, unlike the wild-type protein at any Ca^2+^ concentration or V507D at greater Ca^2+^ concentrations. These results suggest that V507D manifested up to four unfolded EC domains in the absence of Ca^2+^. For both the native dimer and V507D, as the Ca^2+^ concentration decreased, the state space explored by the protein increased, as expected from the stabilizing effect of Ca^2+^.

### Effect of Ca^2+^ Concentration on Refolding.

At the start of a force-ramp cycle after a large unfolding event, the protein sometimes did not return to the starting position that it had occupied at the outset of the previous cycle: The protein either did not refold at all or refolded only partially within the 2 s intercycle resting period. To discern any patterns in the refolding ability of PCDH15 for each construct and Ca^2+^ concentration, we compared the highest state accessed in each cycle with the state of the first segment of the subsequent cycle. If the first segment of the next cycle was in state 1 or 2, we considered this cycle to be a full refolding because the average *x_E_* values in state 1 or 2 were not consistent with the complete unfolding of any EC or PICA domain. We then analyzed the percentage of cycles with full refolding across all Ca^2+^ concentrations.

For the wild-type protein, we found that at a saturating level of Ca^2+^ PCDH15 never unfolded beyond state 2, so we obtained no refolding data for this condition. At a physiological Ca^2+^ concentration, PCDH15 refolded back to state 1 or 2 in 84.2 ± 15.8% (mean ± SEM, *N* = 4 datasets) of the instances. In the absence of Ca^2+^, however, PCDH15 refolded back to state 1 or 2 only 62.9 ± 10.5% (*N* = 6 datasets) of the time (Table 1).

**Table 1.** Extent of full refolding by construct and condition

Construct	[Ca^2+^]	Percentage of full refolding
Wild type	3 mM	—
	20 μM	84.2 ± 15.8
	0 M (1 mM EDTA)	62.9 ± 10.5
V507D	3 mM	82.2 ± 5.7
	20 μM	59.3 ± 8.0
	0 M (1 mM EDTA)	24.0 ± 16.2

The percentage of cycles in which the dimer refolded fully are those that returned to state 1 or state 2 by the beginning of the subsequent cycle after an excursion to some higher state in the previous cycle. The wild-type PCDH15 at a saturating level of Ca^2+^, 3 mM, did not visit any state higher than state 2, so there are no data on complete refolding for that condition. The results are given as mean ± SEMs.

For the V507D mutant at a saturating level of Ca^2+^, PCDH15 fully refolded on 82.2 ± 5.7% (*N* = 24 datasets) of the occasions. At a physiological level of Ca^2+^, this value decreased to 59.3 ± 8.0% (*N* = 16 datasets) in stark contrast with the wild-type protein, for which refolding to state 1 or 2 occurred in most instances at the same concentration of Ca^2+^. Finally, in the absence of Ca^2+^, PCDH15 V507D refolded to state 1 or state 2 in only 24.0 ± 16.2% (*N* = 4 datasets) of the cases, which signified a severe reduction in refolding ability compared to the native protein. The differences in refolding rates between wild-type and V507D dimers suggest that the point mutation disrupts the refolding ability of PCDH15, for V507D dimers unfolded more often than wild-type dimers and refolded properly less often.

### Linear Stiffness of PCDH15.

We calculated the enthalpic stiffness for PCDH15 and PCDH15 V507D by determining the inverse slope of the displacement-force relationship for every cycle in the high-force regime—above 30 pN, the force at which the relationship between force and extension became essentially linear—and averaging the values across all data for each Ca^2+^ condition (*SI Appendix, Note S7*). In 587 determinations at physiological Ca^2+^ levels, PCDH15 had an average enthalpic stiffness of 6.4 ± 0.4 mN m^−1^ (mean ± SEM). If we assume that CDH23, which is approximately 2.3 times as long as PCDH15, has mechanical properties similar to those of PCDH15, its stiffness would be about 2.8 mN m^−1^. For both proteins in series, the stiffness *K*_TL_ of the tip link can then be calculated as

[2] $$\frac{1}{K_{\mathrm{TL}}}=\frac{1}{K_{PCDH15}}+\frac{1}{K_{CDH23}}.$$

**Fig. 5.**
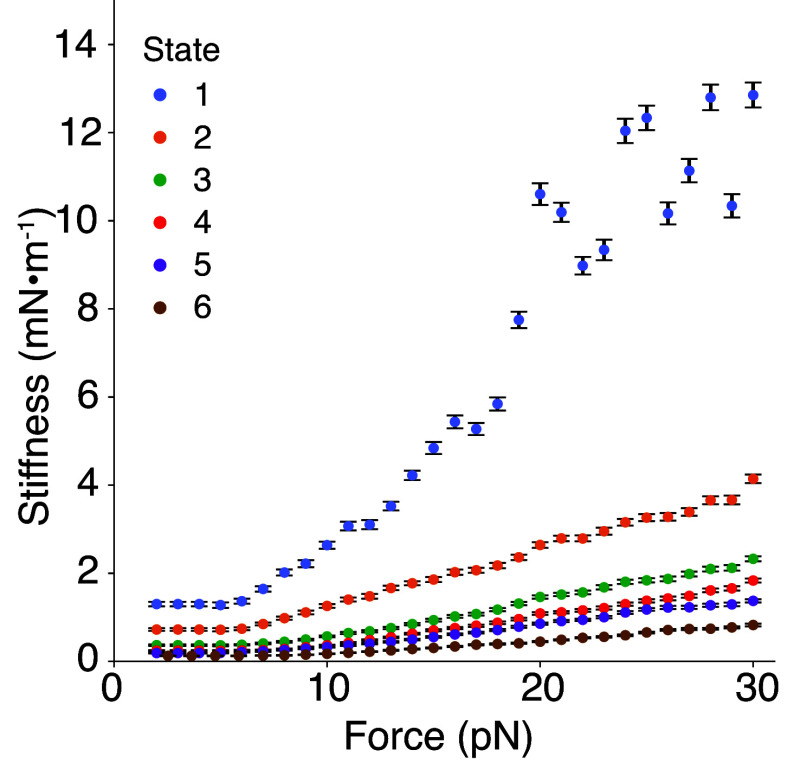
Entropic stiffness of PCDH15’s states as a function of force. The stiffnesses of the states were calculated by determining the inverse of the slope of each displacement-force relationship. In state 1, when PCDH15 was fully folded, the stiffness approached the enthalpic values above 15 pN of applied force. In states 2 to 6, in which PCDH15 had some unfolded portions, the stiffness of PCDH15 remained below the enthalpic limit. State 1 largely comprises trajectories of the wild-type protein at a saturating level of Ca^2+^, whereas the remaining states predominantly contain trajectories at lower Ca^2+^ concentrations and from the V507D mutant. The results suggest that, under physiological conditions, the effective stiffness of PCDH15 is lower than its enthalpic limit of 40 mN m^−1^ at a saturating concentration of Ca^2+^ (*SI Appendix, Note S7*). Error bars represent SEMs.

For the values above, the enthalpic stiffness of the entire tip link in a normal animal is expected to be about 1.9 mN m^−1^. Measurements have shown the stiffness of the gating spring to be between 0.5 mN m^−1^ and 4 mN m^−1^, depending on the characteristic frequency along the cochlea (16), so our value for the enthalpic stiffness of PCDH15 lies within the expected stiffness range for the gating spring. Furthermore, the entropic stiffness of each state is less than the calculated enthalpic stiffness (Fig. 5), which implies that PCDH15 is softer over the range of physiological forces than previously thought. Because the entropic stiffness of each state is lower than the calculated enthalpic stiffness, entropic elasticity dominates the mechanical response of PCDH15 over much of the range of physiological forces.

## Discussion

Our findings indicate that PCDH15, in the dimeric form in which it exists within the cochlea, has the appropriate mechanical properties to serve as a portion of the gating spring. Furthermore, at the physiological Ca^2+^ concentration and over the relevant force range, unfolding of entire EC domains is not the primary response of PCDH15 to force. Instead, the response comprises a series of smaller events that likely stem from the extension of the interdomain linkers and possibly parts of EC or PICA domains.

The Ca^2+^ concentration within the endolymph ranges from approximately 20 μM at the cochlear base to 40 μM at the apex (21). Furthermore, the local depletion of Ca^2+^ might be significant near an open transduction channel: At a distance of 7 nm, calculations indicate that the concentration of Ca^2+^ could fall to half its maximal value. The length of a folded EC domain (30) is about 4.5 nm, so EC10, EC11, and the PICA domain could experience a significantly lower Ca^2+^ level: The behavior of PCDH15 in the distal domains might well lie between the results for physiological levels of Ca^2+^ and those in the ion’s absence. In particular, for a Ca^2+^ concentration of 0 to 20 μM Ca^2+^, PCDH15 could extend around 20 to 100 nm. The most extreme tip link extensions observed experimentally (38) are around 120 nm, but because mechanotransduction is very sensitive—even hair bundle deflections of 1 nm can produce a response—it is likely that the tip link ordinarily extends much less.

In the experiments presented here, we maintained a force of 1 pN between successive force ramps, but future studies could explore how increasing the interramp resting force affects the mechanics of PCDH15. For example, at higher resting tensions, PCDH15 might exhibit a decreased ability to refold or take longer to refold than at a resting tension of only 1 pN.

**Fig. 6.**
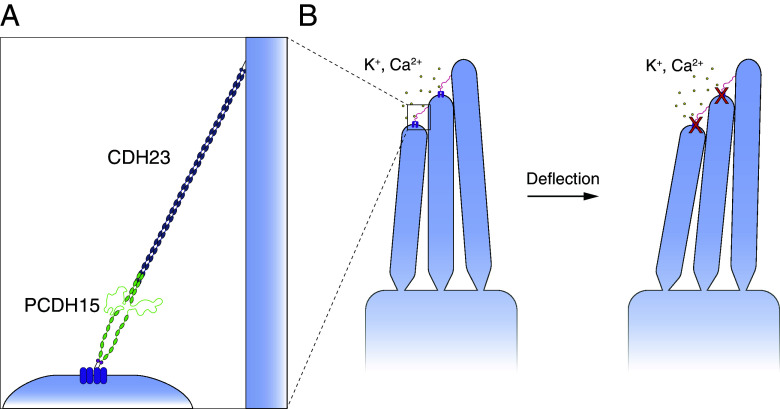
Proposed mechanism of deafness for mutated V507D. (*A*) Our data suggest that V507D is misfolded or partially unfolded over the physiological range of forces experienced in the cochlea. (*B*) If the tip links lack sufficient tension to open the mechanosensitive ion channels when stimulated, the ensuing deficit of Ca^2+^ in the stereocilia causes their degeneration.

The V507D mutation manifests significant unfolding even at a saturating concentration of Ca^2+^, and unfolds to a still more striking extent in the absence of Ca^2+^. Because the end-to-end distance at low force is far greater for the mutant dimer than for the native dimer, V507D is misfolded even at the baseline of 1 pN force. When the applied force is low, such as during the resting period between force-ramp cycles, the two partially unfolded strands of the dimer might tangle with each other, resulting in a misfolded protein that extends easily when force is reapplied. This behavior might ensue because EC5 is more prone to unfolding due to the location of the mutation, which could then predispose the neighboring EC domains to unfold by disrupting the stability of the interdomain linkers. Furthermore, the refolding ability of V507D is significantly lower than that of the native protein at physiological and no Ca^2+^ concentrations for which the comparison is possible. If V507D is unable to refold on a timescale appropriate for normal hearing, on the order of microseconds to milliseconds, a more compliant PCDH15 dimer might underlie a softer overall tip link (Fig. 6).

Insufficient tension applied to the mechanotransduction channels might constitute the mechanism of deafness in people with this and other tip-link mutations. More specifically, without an influx of Ca^2+^ through transduction channels, a hair bundle undergoes remodeling, resulting in shorter stereocilia with abnormal tip shapes (39). Moreover, if transduction is abolished in inner hair cells, their innervation changes: They become reinnervated by inhibitory efferent neurons, which normally contact inner hair cells only before the onset of hearing (40). If V507D cannot convey the appropriate tension to transduction channels and thus prevents their opening, changes in stereociliary structure and synaptic connectivity could underlie deafness.

Although the V507D point mutation disrupts the mechanics of PCDH15, individuals with this mutation retain normal equilibrium and vision, despite the expression of PCDH15 in the vestibule and eye. It is possible that the higher concentration of Ca^2+^ in the vestibular labyrinth allows mutated PCDH15 to function adequately, whereas the lower concentration of Ca^2+^ in the cochlea precludes this. It is also plausible that the mutated protein can function adequately at the lower frequencies, less than 20 Hz, characteristic of the vestibular system (41), but cannot perform well at the higher frequencies, up to 20 kHz, detected by the cochlea (42). PCDH15 V507D might be able to refold on the timescale of low-frequency stimulation in the vestibular system, but not at the higher frequencies experienced in the cochlea. As a result, it is likely that the mutated PCDH15 cannot transmit the appropriate tension to the mechanotransduction channel, resulting in deafness in individuals carrying this mutation. A similar mechanism might underlie the hearing loss associated with many other mutations (17) in PCDH15 and CDH23.

Because PCDH15 consists of repeating structural motifs, it is difficult to ascertain the specific origin of any unfolding event. It seems likely that, once a particular part of the molecule has unfolded, the neighboring regions become more vulnerable to unfolding, especially when Ca^2+^-binding sites are disrupted. The Ca^2+^ ions are coordinated by residues both in the linkers and at the edges of the neighboring domains, so unfolding of a neighboring domain could disrupt one or more binding sites and liberate Ca^2+^. The loss of the bound Ca^2+^ would then predispose the region to further unfolding. Although reducing the force on the protein might allow refolding to occur, proper refolding might become impossible after excessive unfolding.

The experimental conditions imposed some limitations on this study. In vivo, force is applied to PCDH15 through its interaction with CDH23 at a heterotetrameric bond, in which the two N-terminal domains of PCDH15 bind the two N-terminal domains of CDH23. At the C-terminal end of PCDH15, the protein is coupled to the mechanotransduction channel complex by way of LHFPL5, which interacts with the cytoplasmic domain of PCDH15 (31). In our experiments, force was applied to molecular tags attached in series to the ends of PCDH15—to EC1 and to the PICA domain—so we could not precisely replicate the application of force to PCDH15 through the handshake interaction and the cytoplasmic domain of PCDH15.

These results confirm that PCDH15 has the appropriate stiffness to form a component of the gating spring and that its physical properties can be modulated by Ca^2+^. In the case of a hearing-loss mutation, PCDH15 unfolds much more frequently, is softer than the wild-type protein, and has impaired refolding ability, three features that would likely result in inappropriate tension conveyed to the transduction channels in vivo. The findings concerning the V507D hearing-loss mutation underscore how the tension conveyed to transduction channels is critical for normal hearing.

## Methods

Detailed methods are provided in *SI Appendix, Materials and Methods*.

As described previously (17, 18), spherical glass pedestal beads 1.5 µm in diameter were chemically attached to the derivatized surface of a coverslip. Each pedestal bore on its surface a small number of PCDH15 constructs that had been secured at their carboxyl termini, which included SpyTag sequences, to SpyCatcher molecules covalently bound to the bead.

At the outset of an experiment, the surrounding solution was filled at a low density with spherical latex beads 1 µm in diameter and previously coated with streptavidin molecules. One bead—designated the probe—was captured by a position-sensing laser beam with a wavelength of 1,064 nm. With a power of 2 mW at the focal plane, the trapping stiffness of this beam was only 0.007 mN m^−1^, so it produced a force on the probe of 1 pN or less. The beam was nonetheless able to restrain the bead while the experimenter held its center 750 nm above the coverslip, brought it near the pedestal, and observed its Brownian motion on a quadrant photodetector. The thermal motion was suppressed when a tether formed through the interaction of a streptavidin molecule on the probe with a biotin moiety at the amino terminus of the PCDH15 construct.

After a suitable tether had formed, displacement of the stage holding the experimental chamber centered the probe 80 nm closer to the pedestal than the optical axis of the positioning-sensing beam. The experimenter next activated a second beam of laser light with a wavelength of 852 nm and an intensity controlled by a Pockels cell modulator. This beam served as an optical trap with a stiffness adjustable up to 0.3 mN m^−1^. The optical axis of this force-producing beam was situated 200 nm beyond that of the position-sensing beam and in the direction opposite the pedestal. As a consequence, the probe’s position was subsequently determined by two forces: the force exerted by the 852 nm beam and that produced by the PCDH15 tether. When the strength of the force-producing beam was increased, the probe bead was pulled farther from the pedestal; the quadrant photodetector provided a calibrated indication of the displacement along the *X*-axis that ran through the centers of the two beads. This arrangement allowed investigation of the relationship between applied force and molecular extension.

## Supplementary Material

Appendix 01 (PDF)

## Data Availability

All original data are available in a Dryad repository at https://doi.org/10.5061/dryad.stqjq2c8s (43). The custom-written Python programs used for data analysis are available on GitHub at https://github.com/cvillasante/VDCH-2023 (44).
